# Cluster B versus Cluster C Personality Disorders: A Comparison of Comorbidity, Suicidality, Traumatization and Global Functioning

**DOI:** 10.3390/bs12040105

**Published:** 2022-04-12

**Authors:** Laura Y. Massaal-van der Ree, Merijn Eikelenboom, Adriaan W. Hoogendoorn, Kathleen Thomaes, Hein J. F. van Marle

**Affiliations:** 1 GGZ in Geest Mental Health Care, 1081 HJ Amsterdam, The Netherlands; l.massaalvanderree@ggzingeest.nl (L.Y.M.-v.d.R.); m.eikelenboom11@amsterdamumc.nl (M.E.); aw.hoogendoorn@amsterdamumc.nl (A.W.H.); 2 Department of Psychiatry, Amsterdam UMC Location Vrije Universiteit Amsterdam, Boelelaan 1117, 1007 MB Amsterdam, The Netherlands; kathleen.thomaes@sinaicentrum.nl; 3 Amsterdam Public Health, Mental Health Program, 1007 MB Amsterdam, The Netherlands; 4 Sinai Centrum, Arkin Institute for Mental Health, 1033 NN Amsterdam, The Netherlands; 5 Amsterdam Neuroscience, Mood, Anxiety, Psychosis, Stress & Sleep Program, 1007 MB Amsterdam, The Netherlands

**Keywords:** personality disorders, (childhood) traumatization, comorbidity, suicidality, global functioning

## Abstract

A general clinical assumption states that cluster B personality disorders (PDs) represent a more severe form of PD than cluster C PDs. Consequently, most PD research is centered on cluster B PDs (especially borderline PD). Yet, prevalence ratings of cluster C PDs exceed those of cluster B PDs. In this explorative, cross-sectional study, we compared cluster B and C PD patients (*N* = 94) on a wide range of clinically-relevant severity measures, including comorbidity, suicidality, (childhood) traumatization and global functioning. Results showed that, although cluster B PD patients suffered more often from substance use disorders and lifetime suicide attempts, no difference could be established between groups for all other severity measures, including trauma variables. In our study, we additionally included a group of combined cluster B and C PDs, who were largely similar to both other groups. Although our study is insufficiently powered to claim a significant non-difference, these findings emphasize that high rates of comorbidity, suicidality, childhood traumatization and functional impairment apply to both cluster B and C patients. As such, our findings encourage more research into cluster C PDs, ultimately leading to more evidence-based treatments for this prevalent patient group. In addition, the high level of traumatization across groups calls for a routine trauma screening, especially since PD treatment may benefit from concurrent trauma treatment.

## 1. Introduction

Personality disorders (PDs) are a group of severe mental disorders characterized by enduring dysfunctional patterns of cognition, affect regulation, interpersonal functioning and impulse control. These patterns are pervasive across a broad range of personal and social situations and cause considerable personal distress [1]. PDs constitute an important public health problem with respect to the associated functional impairment and extensive use of mental and general health care [2,3]. Prevalence estimates in western societies range from 6% in the general population [4] to as much as 45% in psychiatric outpatients [5]. The *Diagnostic and Statistical Manual of Mental Disorders* [1] categorizes the PDs into three clusters: cluster A (the “odd/eccentric” cluster) includes the paranoid, schizoid and schizotypical PD; cluster B (the “dramatic/emotional/impulsive” cluster) includes the borderline, narcissistic, antisocial and histrionic PD; and cluster C (the “anxious/fearful” cluster) includes the avoidant, dependent and obsessive-compulsive PD. Although these clusters are in part theoretical constructs, this categorization is widely used in clinical practice and treatment programs are constructed in line with PD clusters.

A general clinical assumption is that cluster C PDs represent a less severe form of PD than cluster B PDs. Yet, very few studies have directly compared patients with cluster B PDs to patients with cluster C PDs. By far, the majority of (clinical) research within the field of PDs has been centered on cluster B PDs only and specifically on borderline personality disorder (BPD). As a result, most evidence-based treatments for PDs have only been studied in the context of cluster B PDs [6,7] (but see [8]) and clinical guidelines mostly apply to BPD [9,10]. Prevalence ratings of cluster C PDs however, exceed those of cluster B PDs (2.7% vs. 1.5%) [4] and cluster C patients frequently seek treatment [11]. This makes a direct comparison between cluster B PDs and cluster C PDs clinically relevant.

Existing studies that do compare cluster B and C PDs often focus their reported analysis on a single outcome. In a study by McGlashan et al., for instance, BPD patients are shown to have more lifetime Axis-I diagnoses (particularly PTSD and substance use disorders) than several cluster C PDs [12]. Lenzenweger et al. furthermore report higher odds ratios for general comorbidity in cluster B than cluster C PDs [13]. With respect to suicidality, cluster B—and not cluster C—PDs are shown to be uniquely associated with future suicide attempts [14,15]. Childhood traumatization (CT) is highly prevalent in both cluster B and C PDs [16,17,18,19,20] and is related to a less favorable course of disease in PD and a negative treatment outcome [21,22,23,24] (but see [25]). Although different forms of childhood adversity seem particularly prevalent in cluster B PD patients [18,26], direct comparisons between clusters B and C are rare. In addition, CT in these studies is often assessed only with a self-rated, retrospective questionnaire, presumably producing less reliable results than a structured clinical interview [27]. Finally, cluster B and C PDs have been compared with respect to global functioning [2,28,29]. Most of the studies comparing cluster B and C PDs originate from the Collaborative Longitudinal Personality Disorders Study (CLPS) [30]. This rich cohort prospectively and repeatedly compares four DSM-IV personality disorders, being schizotypal PD (STPD, as a representative for cluster A), BPD (as a representative for cluster B), avoidant PD (as a representative for cluster C), and OCPD (as separable from the other three clusters). Studies coming from this cohort, however, tend to either focus solely on BPD or compare BPD with the (combined) other PDs (including STPD) as a general control group.

In conclusion, although cluster C PDs are highly prevalent, research in PDs is dominated by cluster B PDs (especially BPD) and a clean, direct comparison between the two clusters remains rare. Therefore, the aim of this explorative study is to compare patients with cluster B PDs to patients with cluster C PDs on a wide range of clinically relevant severity measures that have previously been studied primarily in isolation, being comorbidity, suicidality, (childhood) traumatization (including PTSD diagnosis and severity) and global functioning. In contrast to previous literature, we thereby assess CT both through structured clinical interviews and a self-report questionnaire. PDs are diagnosed through a structured clinical interview as well, and analyses only include data on full PD diagnosis, as opposed to mere PD symptoms. In addition, the clinically common and relevant group of patients diagnosed with both a cluster B and cluster C PD (at least one diagnosis of each) is added to the analysis as a separate group.

## 2. Materials and Methods

### 2.1. Participants

The current study included patients who got primarily diagnosed with PD while seeking treatment in a specialized outpatient department for personality disorders of GGZ inGeest Mental Health Hospital in Amsterdam, The Netherlands. This department treats moderate to severe PDs with a wide range of psychotherapeutic interventions such as Schema-Focused Therapy (SFT), Mentalization-Based Treatment (MBT) and Dialectical Behavioral Therapy (DBT). Patients were asked to participate during an intake procedure after they were referred by general practitioners or other mental health institutes for further assessment and/or treatment. General inclusion criteria consisted of (1) Age between 18 and 65 years; (2) Being able to speak, write and understand Dutch sufficiently. General exclusion criteria consisted of: (1) Currently suffering from a comorbid manic or psychotic episode; (2) A (suspected) diagnosis of autism spectrum disorder (ASD) (due to the significant symptomatic overlap between PD and ASD); (3) Mental retardation.

Over the course of data collection (June 2015–July 2016), 203 patients were referred to the outpatient department. Of the 175 patients who met our inclusion criteria, 120 signed informed consent (69%). The 55 patients who opted not to participate did so because they could not be motivated for participation, could not be reached after the first day or gave no specific reason for refusal. For the purpose of this analysis we included only patients with a cluster B and/or C PD (*N* = 94) (DSM-IV: 301.4, obsessive-compulsive; 301.6, dependent; 301.82, avoidant; 301.5, histrionic; 301.81, borderline; 301.83, narcissistic; 301.70, antisocial; 301.0). Patients diagnosed with a PD not otherwise specified (and that were not also diagnosed with a full cluster B or C PD) were excluded.

### 2.2. Design and Fieldwork Procedure

The data were collected cross-sectionally as part of a standard intake procedure and an additional set of self-rating questionnaires were filled in by the patients at home. Over the course of two intake visits to the outpatient department the diagnosis of PDs, PTSD and other Axis I disorders were assessed using semi-structured interviews, as well as the occurrence of CT (see Measures). After intake, participants received an additional set of self-rating research questionnaires probing (among others) severity of childhood trauma, which they filled in at home and sent back in a return envelope. Interviewers were all experienced therapists who were trained extensively in administering the clinical interviews prior to this study.

### 2.3. Measures

PDs were diagnosed using the SIDP-IV (Structured Interview for DSM-IV Axis II Personality Disorders), a semi-structured interview assessing DSM-IV personality disorders [31] (Dutch translation by [32]).

The severity measures analyzed in this study included comorbidity, suicidality, (childhood) traumatization and global functioning. Comorbidity was operationalized as having a comorbid Axis I disorder (apart from PTSD) as measured with the MINI-Plus 5.0.0 (Mini International Neuropsychiatric Interview) [33]. For this study, the following Axis I disorders were assessed through structured interviews: mood disorders (major depressive disorder, dysthymia and bipolar disorder), anxiety disorders (panic disorder, agoraphobia, social phobia, specific phobia, obsessive-compulsive disorder and general anxiety disorder), eating disorders (anorexia and bulimia), substance abuse and dependency, psychotic disorder, somatization disorder, hypochondria, body dysmorphic disorder and pain disorder. The total number of Axis I disorders resembles the sum of all these disorders.

Suicidality was operationalized as currently having a medium to high suicide risk and the amount of lifetime suicide attempts, both measured with the MINI-Plus 5.0.0.

Trauma variables included current PTSD diagnosis, PTSD severity and occurrence and severity of CT. Current PTSD was diagnosed using the PTSD Symptom Scale Interview (PSS-I), a semi-structured interview assessing PTSD symptoms according to DSM-IV criteria [34] (Dutch translation by [35]). PTSD severity scores were based on the sums of the raw PSS-I items. Of the 94 participating patients, 26 did not experience an A criterion trauma according to the DSM-IV, in which case the PSS-I was not administered and no severity score was calculated. The occurrence of CT was measured using the STI (Structured Trauma Interview) [36], which assesses different forms of childhood trauma and adult traumatic experiences through semi-structured interviews. In this study, the occurrence of CT (yes or no) was operationalized as having experienced physical abuse, sexual abuse or both before the age of 16. The STI in addition assessed also other traumatic events before the age of 16 such as the loss of a loved one or having experienced a serious accident. The severity of CT was measured using the CTQ (Childhood Trauma Questionnaire), a self-rating, retrospective questionnaire assessing different forms of abuse and neglect during childhood [37] (Dutch translation by [38]. The CTQ consists of 25 questions assessing emotional, physical and sexual abuse and emotional and physical neglect separately. Additionally, it includes a Minimization/Denial scale with three questions, indicating the potential underreporting of maltreatment. Participants responded to each item in the context of “when you were growing up” and answered according to a five-point Likert scale ranging from “never” = 1 to “very often” = 5. In this study, the overall severity of CT was operationalized as the total score of the 25 CTQ items (with a severity score ranging from 25–125). Of the 94 participating patients, 32 did not complete and return the CTQ. The low completion rate was in all probability due to the fact that the CTQ was the last questionnaire to be filled in and was located on the backside of the booklet of questionnaires and therefore often overlooked. In the current sample, the CTQ total score had good reliability (Cronbach’s α = 0.928).

Global functioning variables included relationship functioning, employment and social- and role functioning. Employment (currently having a paid job) and relationship functioning (currently having a partner relationship and total amount of current friends) were assessed using a general biographical questionnaire, assessing demographic information. Social functioning and role functioning were measured using the SF-36 (Short Form (36) Health Survey) [39], a 36-item, self-rating questionnaire assessing patient’s health in 8 domains: vitality, physical functioning, bodily pain, general health perceptions, physical role functioning, emotional role functioning, social functioning and mental health [39]. For this study, we used the scores of the domains social functioning and emotional role functioning (referring to role limitations due to emotional problems). Of the 94 participating patients, 5 did not complete and return the SF-36.

### 2.4. Statistical Analyses

Demographic and clinical characteristics of the total sample, as well as information on the distribution of co-morbidity, suicidality, trauma and global functioning variables, were summarized using descriptive statistics.

To examine in an explorative manner whether patients with a cluster B PD (*N* = 30), patients with a cluster C PD (*N* = 40) and patients with both a cluster B and C PD (*N* = 24) differed from each other in terms of the described severity measures, cross-tabulations were done and tested using Fisher’s exact test for all categorical variables and means and standard deviations were calculated and tested using one-way analysis of variance (ANOVA) for all continuous variables.

Post-hoc analyses were performed to test differences in severity measures between the separate groups (cluster B PD, cluster C PD and cluster B and C PD). Again, cross-tabulations were performed for all categorical variables and ANOVA’s for all continuous variables. These analyses were adjusted using the Bonferroni method for multiple comparisons, correcting for 3 simultaneous tests per analysis.

Because of unequal and low sample sizes of the separate groups, the Fisher’s exact test was used to test independence in cross-tabulations and the Welch’s test was used to test equality of means. All analyses were conducted using IBM SPSS Statistics version 22.0 for Windows.

## 3. Results

### 3.1. Sample Characteristics

Demographic characteristics of the total sample are shown in Table 1. Of the total sample of 94 patients, about two-thirds were female, the mean age was 36.2, Dutch was the primary nationality and the amount of attained educational years corresponded to the middle level.

### 3.2. Clinical Characteristics of Total Research Sample

Table 1 additionally shows the clinical characteristics of the total research sample. With respect to the experimental groups, nearly 32% were diagnosed with a cluster B but no cluster C PD (*N* = 30), mainly BPD. 42.6% were diagnosed with a cluster C but no cluster B PD (*N* = 40), with a relatively even distribution of the different cluster C PDs. About one-fourth of the sample was classified as having both a cluster B and a cluster C PD (*N* = 24), with BPD dominating this subgroup. For prevalence ratings of specific PDs within the experimental groups see Table 1 (reported as a percentage within each experimental group). Note that patients within one experimental group often had multiple PDs within their respective clusters. Of the total research sample (across experimental groups), 57.4% were diagnosed with a cluster B PD, 68.1% with a cluster C PD and 8.5% were additionally diagnosed with a cluster A PD (paranoid PD).

Comorbid DSM-IV Axis I disorders were very common across the total sample, especially mood disorders (almost 60%) and anxiety disorders (56.5%, excluding PTSD). Patients on average had 2.2 (SD = 1.6) comorbid Axis I disorders. Furthermore, suicidality was high with almost 36% of patients exhibiting a medium to high suicide risk at the time of study and almost 32% having attempted suicide sometime in their lives.

With respect to trauma variables, more than 45% of the sample was diagnosed with current PTSD. The mean PTSD severity score was 26.4 (SD = 12.0). In addition, childhood traumatization was highly prevalent in the sample. About two-thirds (63%) experienced physical and/or sexual abuse before the age of 16 years. The mean severity of CT, as measured with the CTQ, was 57.1 (SD = 19.5), corresponding to a moderate to severe level of CT.

Finally, concerning global functioning, approximately 43% of the sample had a relationship and 33% had a paid job at the time of the assessment. On average, participants had six friends. Participants showed low social and emotional role well-being, as indicated by low scores on social functioning and emotional role functioning with mean scores of 40.7 (SD = 27.2) and 28.5 (SD = 35.4), respectively. This indicates severe and frequent limitations during social activities due to health and emotional problems (low social well-being) and problems in work and other daily activities due to emotional problems (low emotional role wellbeing).

### 3.3. Comparison of Comorbidity and Suicidality Variables

Table 2 shows the comparison between the cluster B, cluster C and cluster B and C PD groups on multiple comorbidity measures and suicidality variables. Using cross-tabulations (testing independence with Fisher’s exact tests), we found that the three diagnosis groups scored significantly different on having a current anxiety disorder (*p* = 0.030), being dependent on alcohol and/or drugs in the last 12 months (*p* = 0.021) and having attempted suicide sometime during their lives (*p* = 0.001). Following up on the tests of equality across the three groups, we found in a post-hoc analysis using cross-tabulations (with Fisher’s exact test) that patients with both a cluster B and C PD significantly more often had a current anxiety disorder than patients with a cluster B PD (77% vs. 40%, *p* = 0.011). In addition, recent dependency on alcohol and/or drugs occurred more often in patients with a cluster B PD than in patients with a cluster C PD (37% vs. 10%, *p* = 0.009). Finally, cluster B PD patients significantly more often attempted suicide sometime during their lives than patients with a cluster C PD (53% vs. 13%, *p* < 0.001). The three diagnosis groups did not statistically significantly differ in the total number of comorbid Axis I disorders, having a current mood or somatization disorder and exhibiting a medium to high suicide risk at the time of assessment.

### 3.4. Comparison of Trauma Variables

In comparing the three diagnosis groups with respect to trauma, we found that none of the trauma variables showed statistically significant differences across the three diagnosis groups (Table 3). Numerically, however, current PTSD and physical and/or sexual abuse before the age of 16 did occur more often in patients with a cluster B PD and patients with a cluster B and C PD than in patients with a cluster C PD. PTSD severity, the occurrence of other traumatic events before the age of 16 and severity of CT were very similar between groups.

### 3.5. Comparison of Global Functioning Variables

Using cross-tabulations, we found that the three diagnosis groups scored significantly different on currently having a relationship (*p* = 0.042) (Table 4). In a follow-up analysis, using cross-tabulations, we found that cluster B PD patients were significantly less involved in a partner relationship than patients with a cluster C PD (27% vs. 56%, *p* = 0.016). Using an ANOVA (with Welch test), we similarly show that the number of friends differed significantly between the three diagnosis groups (F (2, 52.9) = 3.396, *p* = 0.041). In follow-up analysis, patients with both a cluster B and C PD had significantly less friends than cluster C PD patients (M = 3.8, SD = 3.7 vs. M = 7.2, SD = 6.9, F (1, 57.7) = 6.199, *p* = 0.016). Differences between groups for having a paid job and on scores for social and emotional role functioning were not statistically significant.

## 4. Discussion

### 4.1. Discussion of Outcomes

In this study, we compared patients with cluster B and C PDs on a wide range of clinically-relevant severity measures, including comorbidity, suicidality, (childhood) traumatization and global functioning. Cluster B PD patients were shown to suffer more often from substance use disorders than patients with a cluster C PD and more often attempted suicide in their lifetimes. In addition, they less often had a partner relationship. On all other severity measures studied, including all trauma variables, we could not establish differences between cluster B and cluster C PD patients. In our study, we additionally included the clinically relevant group of patients with both a cluster B and C PD, who were diagnosed more often than cluster B PD patients with an anxiety disorder and on average had fewer friends than cluster C PD patients.

First, we showed that overall, our sample of treatment-seeking PD patients represents a clinically severe and representative group. In accordance with previous literature, around 60% of our patients suffered from a comorbid mood- or anxiety disorder [4,13], one-third attempted suicide during their lives and one-third exhibited a medium to high suicide risk at the time of the study [14,40]. In addition, almost half of the sample suffered from PTSD and over two-thirds of the sample experienced some form of CT [20,41,42,43,44].

Second, when looking into the difference between groups, we showed that concerning comorbidity, cluster B PD patients suffered more often from dependence on alcohol and/or drugs in the last 12 months than patients with a cluster C PD. This is in line with previous literature showing especially cluster B PDs—and not cluster C PDs—being associated with substance use disorders [45,46,47]. We found no difference between groups in the total number of current comorbid (Axis I) disorders. This seems in contrast to two previous investigations, which mainly showed high (Axis I) comorbidity in BPD patients [12,13]. McGlashan et al. [12], however, compared BPD with AVPD and OCPD (instead of whole PD clusters) in inpatients and concerned lifetime (as opposed to recent/current) Axis I disorders. Lenzenweger et al. [13] in turn, based their PD diagnosis on screening questions (instead of semi-structured, clinical interviews) in a general population sample (as opposed to a sample of treatment-seeking patients). In addition, our sample may have been too small to detect existing differences. Furthermore, we found no difference in the prevalence of mood and anxiety disorders. This contrasts with Gude and Vaglum [48], who reported more mood and anxiety disorders in cluster C than cluster B PD patients, although their sample consisted of in-patients, possibly reflecting a more severe cluster C PD group.

Third, when comparing suicidality, cluster B PD patients considerably more often attempted suicide during their lifetime than patients with a cluster C PD. This is in line with previous literature showing that BPD (and not cluster C PDs) was uniquely related to prospective suicide attempts [14,15] and warrants the focus of some specialized BPD treatment programs, like DBT, on (para)suicidal behavior [49]. Both groups exhibited a comparable medium-to-high risk for suicide at the time of assessment. This may indicate that outside of the number of lifetime suicide attempts, cluster C PD patients exhibit other signs of suicidality, like suicidal ideations or planning suicide.

Fourth, we could not establish differences between cluster B and cluster C PD patients on any of the trauma variables measured. Concerning the prevalence of PTSD, this is in contrast to previous research. McGlashan et al. [12] and Yen et al. [43] found PTSD to be more prevalent in the clinical CPLS cohort in patients with BPD than in patients with AVPD or OCPD, yet not comparing the full range of cluster B and C PDs. Moreover, Amstadter et al. [50] found higher levels of PTSD in individuals with BPD symptoms than in individuals with other (including cluster C) PD symptoms. In their population-based sample, personality pathology was based on PD symptoms rather than a full PD diagnosis. This suggests that our sample of treatment-seeking PD patients represents a more severe group, which may especially hold for the habitually avoidant cluster C PD patients. Again, our sample size may have additionally been too limited to pick up existing differences. The high concurrence previously found between especially BPD and PTSD has led to the suggestion to reconceptualize BPD as a trauma spectrum disorder [51]. Yet, others have proposed a new diagnostic category, known as complex PTSD (CPTSD), to clinically capture the group of patients having experienced prolonged, interpersonal trauma and show both PTSD symptoms and additional problems such as affect dysregulation and dissociation [52]. A cluster analysis of personality characteristics within a group of patients meeting the CPTSD diagnosis found that the relationship between PDs and CPTSD is complex and exceeds just borderline characteristics [53]. The study identified five clinically relevant subtypes of CPTSD that were characterized by different levels of introversion and disinhibition and were thus differently associated with avoidant and dependent personality symptoms on the one hand and borderline and antisocial personality symptoms on the other. Our finding of similarly high levels of PTSD and PTSD severity in cluster C patients may underscore the important role of cluster C personality traits in complex PTSD. With respect to CT, we could not detect differences between groups in the occurrence or severity of CT. Although there is considerable literature relating individual (cluster B and C) PDs to (sometimes specific forms of) CT [16,17,20,54,55], a direct comparison between the two clusters is rare. An exception is the study of Zhang et al., showing that emotional abuse and sexual abuse are more prevalent in cluster B than in cluster C outpatients [19]. This study, like most studies on CT within PDs, used a self-rated, retrospective questionnaire to assess CT. In addition to this, we assessed CT with a structured clinical interview, possibly resulting in a more trustworthy account of CT due to less reporting bias [27]. One could argue that this has resulted in higher reports of CT, especially for habitually anxious and avoidant cluster C PD patients.

Fifth, when comparing global functioning, we found cluster B PD patients to have fewer partner relationships than cluster C PD patients. We did not find evidence for differences in the level of functional impairment between groups, specifically concerning work-related, emotional and social functioning. This corresponds to previous research reporting similar GAF ratings and levels of interpersonal problems for BPD, OCPD, AVPD and dependent PD (DPD) [56], while cluster B PD patients were found to be less often married or cohabitant than patients with cluster C PDs [48,56]. Reports from the CLPS cohort do show less global impairment in OCPD and AVPD patients than in BPD patients [2,28,29]. In these studies, however, patients with both AVPD and BPD were allocated to the BPD group, possibly leading to an amplified difference between the BPD group and the pure AVPD group.

Finally, the present study included a third group of patients with both a cluster B and cluster C PD, since this is a clinically common and relevant group. We found these patients to suffer more often from comorbid anxiety disorders than cluster B PD patients and have fewer friendships than patients with solely a cluster C PD. No differences appeared for the other outcome measures. Although our sample size is too small to claim a non-difference, this result may suggest that having combined PDs in both clusters B and C does not automatically result in a clinically more severe illness. Note that patients in the combined group did not automatically have more PDs in total, as patients in the cluster B and C groups often had multiple PDs within their respective clusters. One of the few existing studies focusing on patients with combined PDs showed that inpatients with a cluster C PD and one or more comorbid PDs from cluster A or B just as often had depressive and anxiety disorders as inpatients with solely a cluster C PD. They did, however, suffer more often from substance use disorders, had a higher number of comorbid Axis I disorders and were less often married or cohabitant [48]. In this comorbid group, however, nearly half of the cases were diagnosed with (cluster A) paranoid PD, possibly accounting for a generally more severe disease state than our combined cluster B and C group.

### 4.2. Strengths and Limitations

The present study has several strengths as well as certain limitations. One particular strength is that we assessed all diagnoses using structured clinical interviews (SIDP-IV for PDs, PSS-I for PTSD and MINI-PLUS for other Axis-I disorders), ensuring a high level of diagnostic validity. In addition, CT was assessed using both a structured clinical interview (STI) and a self-rated, retrospective questionnaire (CTQ). Given the possible reporting bias related to avoidance, especially in the cluster C PD group, this may have resulted in a more trustworthy account of the history of CT than using the CTQ alone [27]. We also used the CTQ to get additional information on the severity of CT. Another strength is the use of mutually exclusive groups, that is patients in the cluster B group did not meet the criterion for a cluster C PD and vice versa. A separate group was added to the analysis for patients with both a cluster B and C PD. This led to a relatively clean comparison between cluster B and cluster C PD patients, which has been difficult to accomplish in existing studies. In addition, the large proportion of male participants (32%) is a strong point of our study given the predominance of female patients in most studies on PDs. Finally, we selected a group of treatment-seeking patients (as opposed to a population-based sample) rendering our results clinically relevant.

Our findings, however, are also subject to several limitations. Our most important limitation is the relatively small sample size, rendering possible less obvious differences between PD cluster groups (Cohen’s d < 0.85 or differences in proportions < 33%) undetected. Reversely, with no difference found between groups on the majority of outcome measures, the sample size is likewise too small to claim that the groups are in fact similar. Including PD patients in research has been proven difficult before, and the participation rate in this study was 69%. The participating patients were very thoroughly evaluated, however, increasing the validity of the data obtained. In addition, our sample of cluster B PD (and combined cluster B and C PD) patients was dominated by BPD patients around 90%. This makes it difficult to extrapolate our findings to all cluster B PDs equally. Our sample did, however, include a limited number of patients with a histrionic, narcissistic and/or antisocial PD. Because this distribution reflects the normal distribution among treatment-seeking outpatients with a cluster B PD, our findings have implications mostly for the cluster B outpatient group encountered in everyday clinical practice. As such, our findings also do not directly extrapolate to clinical populations of PD inpatients or individuals with PD traits in the general population. As described throughout the discussion section, the difference between cluster B and C PDs seems to be more outspoken in clinical samples. There, cluster B PD patients generally show higher severity in comorbidity, trauma variables and global functioning [2,12,28]. Furthermore, the presence of comorbid psychiatric disorders, most notably mood and anxiety disorders, could potentially affect some of the outcome measures, such as present suicide risk or CT severity ratings. Whereas we did measure the presence of several Axis I disorders, we did not measure their severity, preventing a covariate analysis. Finally, it may be worth mentioning that although a multiple-comparison (Bonferroni) correction was applied post-hoc (correcting for three simultaneous tests over groups), we did not correct for the number of tested variables, possibly risking a type I error. Based on the relatively small sample size and explorative nature of the study, we opted to lower the risk of overcorrection instead.

### 4.3. Clinical and Research Implications

Our findings largely corroborate earlier studies showing that cluster B PD patients on a limited number of indicators represent a more severe patient group than cluster C PD patients. Not surprisingly, these indicators coincide with the diagnostic criteria of (most) cluster B PDs, such as impulsivity (e.g., in alcohol/drug use), (para)suicidal behavior and inability to sustain a stable relationship. Moreover, the found differences emphasize the need for specialized treatment programs that focus on reducing self-harming or impulsive behavior that DBT already does. With respect to trauma, our finding of high prevalence ratings in both cluster B and C PDs underscore the need for routine assessment of PTSD diagnosis as well as CT occurrence and severity in both groups of patients. This take-home message seems especially important since the clinical manifestation of prolonged forms of CT often requires additional treatment strategies that can be given before or in parallel to standard PD treatments [21]. Furthermore, a history of CT may have a negative impact on PD treatment [21,22,24] (but also see [25]), often necessitating longer or more intensive forms of psychotherapy.

For the majority of severity measures studied, we found no difference between groups. Although our study clearly lacks sufficient power to directly test a non-difference, these null findings may indicate that, contrary to general clinical assumption, help-seeking cluster C PD patients on some level represent an equally severe and traumatized patient group as patients with a cluster B PD. Together with the high prevalence of cluster C PDs [4], this may encourage more research initiatives focusing on this group of patients as a second take-home message. So far, cluster B PDs and mainly BPD have dominated the research agendas. Hence, most evidence-based treatments for PDs are only proven effective for BPD and clinical guidelines for PDs almost exclusively apply to BPD patients. Initiating new (randomized, controlled) treatment studies testing the effectiveness of existing PD therapies in cluster C PD patients [8,57,58] could be a first step to broadening the therapeutic arsenal and ultimately developing a more PD type-specific clinical guideline.

Finally, a follow-up of our study would benefit from a larger sample size, ideally also including the elusive group of cluster A PD patients. Adding a longitudinal approach could clarify how the different severity measures change over time or respond to treatment, and whether this is different for the different PD groups.

## 5. Conclusions

In sum, using a thoroughly evaluated and representative sample of treatment-seeking PD patients, we show that high rates of comorbidity (including PTSD), suicidality, childhood traumatization and functional impairment apply to both cluster B and C patients. Cluster B PDs do represent a more severe disease category with respect to alcohol and drug use and lifetime suicide attempts, justifying the specialized treatment programs for cluster B PDs that focus on these specific symptoms. For the majority of outcome measures, we found no difference between groups. Although our study is insufficiently powered to claim a non-difference, these findings may represent an initial step towards more research momentum for cluster C PDs, ultimately leading to more evidence-based treatments and disorder-specific guidelines for this prevalent patient group. In addition, the high level of traumatization in both PD groups, calls for routine screening of occurrence and severity of CT and PTSD diagnosis, especially since treatment efficacy in PDs may benefit from preceding or concurrent trauma treatment.

## Figures and Tables

**Table 1:** Demographics and clinical characteristics of the sample (*N* = 94).

Characteristic	*N*	*%* or *M* (*SD*)
Sex		
Male	30	31.9%
Female	64	68.1%
Age (in years)		36.2 (10.3)
Country of birth (The Netherlands)	78	82.6%
Number of years of education attained		12.4 (3.3)
**Axis II personality disorders (SIDP-IV)**		
Cluster A personality disorder(s)	8	8.5%
Cluster B personality disorder(s)	54	57.4%
Cluster C personality disorder(s)	64	68.1%
**Diagnosed with cluster B (no cluster C) personality disorder(s)**	30	31.9%
Borderline personality disorder	26	86.7% *
Histrionic personality disorder	4	13.3% *
Narcissistic personality disorder	3	10.0% *
Antisocial personality disorder	1	3.3% *
**Diagnosed with cluster C (no cluster B) personality disorder(s)**	40	42.6%
Obsessive-compulsive personality disorder	19	47.5% *
Dependent personality disorder	18	45.0% *
Avoidant personality disorder	18	45.0% *
**Diagnosed with both cluster B and C personality disorder(s)**	24	25.5%
Borderline personality disorder	22	91.7% *
Histrionic personality disorder	2	8.3% *
Narcissistic personality disorder	2	8.3% *
Antisocial personality disorder	4	16.7% *
Obsessive-compulsive personality disorder	12	50.0% *
Dependent personality disorder	6	25.0% *
Avoidant personality disorder	13	54.2% *
**DSM-IV axis I disorders (MINI-Plus 5.0.0), excluding PTSD ^1^**		
Amount of axis I disorders (excl. PTSD) per person		2.2 (1.6)
Current mood disorders	55	59.8%
Current anxiety disorder(s), excl. PTSD	52	56.5%
Dependency on alcohol and/or drugs (last 12 months)	19	20.7%
Current somatization disorder(s)	10	11.0%
Present suicide risk medium-high	33	35.9%
Lifetime suicide attempt	29	31.9%
**Trauma variables**		
Current PTSD (PSS-I)	42	45.7%
PTSD severity (PSS-I, range: 0–51)		26.4 (12.0)
Physical and/or sexual abuse < 16 years (STI)	57	63.3%
Other traumatic experiences < 16 years (STI)	70	77.8%
Overall severity of childhood trauma (CTQ, range: 25–125)		57.1 (19.5)
**Global functioning variables**		
Having a partner (biographic questionnaire)	39	42.9%
Number of friends (biographic questionnaire)		6.1 (6.6)
Having a paid job (biographic questionnaire)	29	33.0%
Social role functioning (SF-36, range 0–100)		40.7 (27.2)
Emotional role functioning (SF-36, range 0–100)		28.5 (35.4)

Note. Due to missing values, total *N* varies between 62 (for CTQ) and 94. SIDP-IV = Structured Interview for DSM–IV Personality; MINI = Mini-International Neuropsychiatric Interview for Axis I disorders; PTSD = posttraumatic stress disorder; PSS-I = PTSD Symptom Scale Interview; STI = Structured Trauma Interview; CTQ = Childhood Trauma Questionnaire; SF-36 = Short Form (36) Health Survey. * Percentage within specific experimental group. ^1^ Axis I disorders with percentages below 9% are not reported.

**Table 2:** Comparison of comorbidity and suicidality variables between cluster B, cluster C and cluster B and C PDs (*N* = 94).

	Cluster B (*N* = 30)	Cluster C (*N* = 40)	Cluster B and C (*N* = 24)	Overall *p*-Value ^a^
Number axis I mean (*SD*)	2.2 (1.6)	2.0 (1.4)	2.6 (1.9)	0.360
Current mood disorder %	57%	55%	73%	0.370
Current anxiety disorder % (excl. PTSD)	40% ^b^	58%	77% ^b^	0.030
Current somatization disorder %	13%	10%	10%	0.913
Dependency on alcohol and/or drugs (last 12 months) %	37% ^c^	10% ^c^	18%	0.021
Present suicide risk medium- high %	30%	33%	50%	0.279
Lifetime suicide attempt %	53% ^d^	13% ^d^	36%	0.001

Note. PDs = personality disorders; PTSD = posttraumatic stress disorder. Due to missing values, the total *N* varies between 91 and 94. All variables measured with the MINI-Plus 5.0.0 ^a^, based on Welch test for ANOVA (for continuous variables) or Fisher’s exact test (for dichotomous variables). ^b–d^ Post hoc analyses to communicate significant differences in comorbidity between two groups of patients in univariate analysis, using Bonferroni corrected significance level of 0.05/3 = 0.0167.

**Table 3:** Comparison of trauma variables for cluster B, cluster C and cluster B and C PDs (*N* = 92).

	Cluster B (*N* = 30)	Cluster C (*N* = 40)	Cluster B and C (*N* = 24)	Overall *p*-Value ^a^
Current PTSD % (PSS-I)	45%	36%	63%	0.127
PTSD severity mean (*SD*) (PSS-I sum score)	25.8 (11.8)	26.2 (13.2)	27.4 (10.6)	0.907
Physical and/or sexual abuse < 16 years % (STI)	70%	53%	73%	0.215
Other traumatic experience < 16 years % (STI)	77%	78%	79%	1.000
Overall severity childhood trauma mean (*SD*) (CTQ)	59.7 (18.5)	56.1 (21.9)	55.3 (15.6)	0.739

Note. Due to missing values, the total *N* varies between 62 (for CTQ) and 92. PSS-I = PTSD Symptom Scale Interview; PTSD = posttraumatic stress disorder; STI = Structured Trauma Interview; CTQ = Childhood Trauma Questionnaire ^a^ Based on Welch test for ANOVA (for continuous variables) or Fisher’s exact test (for dichotomous variables).

**Table 4:** Comparison of global functioning variables for cluster B, cluster C and cluster B and C PDs (*N* = 91).

	Cluster B (*N* = 30)	Cluster C (*N* = 40)	Cluster B and C (*N* = 24)	Overall *p*-Value ^a^
Having a relationship % (biographic questionnaire)	27% ^b^	56% ^b^	41%	0.042
Number of friends mean (*SD*) (biographic questionnaire)	6.3 (7.7)	7.2 (6.9) ^c^	3.8 (3.7) ^c^	0.041
Paid job % (biographic questionnaire)	35%	35%	27%	0.843
Social functioning mean (*SD*) (SF-36)	46.7 (30.6)	41.1 (25.8)	31.5 (22.9)	0.129
Emotional role functioning mean (*SD*) (SF-36)	34.4 (39.6)	25.4 (33.3)	25.4 (33.2)	0.572

Note. Due to missing values, total *N* varies between 87 and 91. SF-36 = Short Form (36) Health Survey; with lower scores indicating more limitations, ^a^ based on Welch test for ANOVA (for continuous variables) or Fisher’s exact test (for dichotomous variables). ^b,c^ Post hoc analyses to communicate significant differences (after Bonferroni correction) in global functioning between two groups of patients in univariate analysis, using Bonferroni corrected significance level of 0.05/3 = 0.0167.

## Data Availability

Data available on request due to restrictions e.g., privacy or ethical.
